# Enhanced oral bioavailability and anticancer efficacy of fisetin by encapsulating as inclusion complex with HPβCD in polymeric nanoparticles

**DOI:** 10.1080/10717544.2016.1245366

**Published:** 2017-02-03

**Authors:** Amrita Kadari, Sagarika Gudem, Hitesh Kulhari, Murali Mohan Bhandi, Roshan M. Borkar, Venkata Ramana Murthy Kolapalli, Ramakrishna Sistla

**Affiliations:** 1 Medicinal Chemistry & Pharmacology Division, CSIR-Indian Institute of Chemical Technology, Hyderabad, India,; 2 National Centre for Mass Spectrometry, Indian Institute of Chemical Technology, Hyderabad, India, and; 3 Department of Pharmaceutical Sciences, A.U. College of Pharmaceutical Sciences, Andhra University, Visakhapatnam, India

**Keywords:** Fisetin, hyroxypropyl β-cyclodextrin, inclusion complex, PLGA nanoparticles, anticancer activity, oral bioavailability

## Abstract

Fisetin (FST), a potent anticancer phytoconstituent, exhibits poor aqueous solubility and hence poor bioavailability. The aim of the present study is to improve the oral bioavailability of FST by encapsulating into PLGA NPs (poly-lactide-co-glycolic acid nanoparticles) as a complex of HPβCD (hydroxyl propyl beta cyclodextrin) and to assess its anti-cancer activity against breast cancer cells. FST-HPβCD inclusion complex (FHIC) was prepared and the supramolecular complex formation was characterized by FTIR, DSC, PXRD and ^1^H NMR. FHIC encapsulated PLGA nanoparticles (FHIC-PNP) were prepared and were studied for *in vitro* anticancer activity, cellular uptake, apoptosis and reactive oxygen species generation in MCF-7 human breast cancer cells. Comparative bioavailability of FST was determined after oral administration in C57BL6 mice as pure FST and FHIC-PNP. The results revealed that FHIC-PNP not only enhanced the anti-cancer activity and apoptosis of FST against MCF-7 cells but also improved its oral bioavailability, as demonstrated by increased peak plasma concentration and total drug absorbed.

## Introduction

Among the phytoconstituents, compounds from flavonoid family have shown excellent pharmacological properties. One such flavonoid is Fisetin, 2-(3,4-dihydroxyphenyl)-3,7-dihydroxychromen-4-one) (FST), present in fruits and vegetables, that possesses several biological properties in the prevention and treatment of various diseases including cancer. FST has been found to be cytotoxic toward various cancers viz. breast (Noh et al., 2015) prostate (Adhami et al., 2012), liver (Maurya & Trigun, 2016), colon (Suh et al., 2009), lung (Kang et al., 2015), neuroblastoma (Yi et al., 2014), human cervical cancer (Chou et al., 2013) and teratocarcinoma (Tripathi et al., 2011). Additionally, FST is documented for anti-angiogenic activity against Lewis lung carcinoma cells (Bhat et al., 2012). However, the therapeutic applications of FST have been hampered due to its poor aqueous solubility (10.45 μg/ml) (Bothiraja et al., 2014) and low oral bioavailability (44.1%) (Seguin et al., 2013). Further, FST also undergoes extensive first pass metabolism, P-glycoproteins (p-gp)-mediated efflux and enzymatic degradation in gastrointestinal tract upon oral administration.

Nanotechnology-based delivery systems have emerged as potential vehicles for the delivery of drugs, phytoconstituents and nucleic materials (Mudshinge et al., 2011; Pooja et al., 2014). Previously, attempts have been made for the delivery of FST using lipid-based carriers and chelating agents such as calcium (Bothiraja et al., 2014). However, these attempts were limited to intraperitoneal and intravenous administration only. Moreover, these nanoformulations were developed using native lipophilic FST, resulting into low encapsulation efficiency (EE) with respect to nanocarrier matrix.

Herein, we have prepared FST-hydroxyl propyl beta cyclodextrin (HPβCD) inclusion complex (FHIC) to increase the aqueous solubility of FST followed by incorporation in polymeric-based nanoparticles. Cyclodextrin derivatives are well known for their ability to enhance the solubility of poorly water soluble compounds. Additionally, cyclodextrins inhibit the p-gp, responsible for drug efflux and cytochrome P450, responsible for drug metabolism that assists in enhanced oral bioavailability. Among cyclodextrin derivatives, HPβCD has the highest drug solubilizing capability along with least toxicity (Dua et al., 2014; Madan et al., 2014).

Poly-lactic-co-glycolic acid (PLGA) is a Food and Drug Administration (FDA)-approved, biocompatible and biodegradable polymer and has been widely exploited for encapsulating anticancer drugs (Sah et al., 2013; Kulhari et al., 2015). In addition, drug release rates, size and loading can be easily manipulated to provide further control over drug delivery (Zhao & Feng, 2010). Like other nanosystems, PLGA nanoparticles (PNP) are also taken up by enterocytes and specialized M cells overlaying Peyer’s patches in the small intestine that results into bypassing of CYP 450 mediated metabolism, p-gp mediated efflux and hepatic first pass metabolism of drugs.

Hence, the present investigation aims to design nanoparticles with two basic objectives. The first objective was to develop FHIC to increase the solubility of poorly soluble FST and its loading capacity in the nanoparticles. In the second objective, we aimed to incorporate FHIC into PLGA nanoparticles (FHIC-PNP) to provide controlled drug release and to improve oral bioavailability of FST.

## Experimental

### Materials and methods

Poly(lactide-co-glycolide) PLGA 50:50 (Resomer® RG 502), FST, Pluronic F-68, HPβCD, d-α-Tocopherol polyethylene glycol 1000 succinate (TPGS), Hoechst 33258 and Annexin V-FITC apoptosis detection kit were obtained from Sigma Aldrich (St. Louis, MO). MCF-7 cell lines were obtained from American Type Culture Collection (ATCC, Manassas, VA). Dulbecco’s modified eagle medium (DMEM) medium, Trypsin EDTA solution and fetal bovine serum (FBS) were purchased from Gibco, Gran Island, NY. All other chemicals and solvents were of analytical grade.

### Phase solubility analysis

To investigate the phase solubility of FST with or without HPβCD, an amount of 5 mg FST was dispersed in 10 ml of PBS 7.4 containing various concentration of HPβCD (2–16 mM). The samples were stirred using an orbital shaker at 200 rpm and maintained at a temperature of 37 ± 1 °C for 48 h (Qiu et al., 2014). The samples were filtered through 0.22 μm membrane filters (Millipore, Darmstadt, Germany) and analyzed using UV visible spectrophotometer (Lambda 25, Perkin Elmer, Waltham, MA) at 360 nm. The apparent stability constant for the FHIC was calculated from the slope according to the following equation: *K*_C_=Slope/*S*_0_ (1 − Slope), where *K*_C_ is the apparent stability constant and *S*_0_ is the solubility of drug in absence of HPβCD. The experiments were performed in triplicate.

### Preparation of FHIC solid complex

FHIC in 1:1 ratios (mM) of FST with HPβCD were prepared by simple coacervation technique (Hu et al., 2012). HPβCD was dissolved in ethanol and stirred at 50 °C for 30 min. FST was added to the ethanolic solution of HPβCD and was allowed to stir further for 1 h. The organic solvent was evaporated under reduced pressure in a rotary evaporator at 50 °C and vacuum dried to produce FHIC complexes. The inclusion complex was dissolved in distilled water to obtain a clear solution for further use. The physical mixture of FST and HPβCD (1:1 mM ratio) were prepared by thoroughly mixing the two components in a mortar for 10 min.

### Determination of apparent solubility of the FST and FHIC in aqueous phase

For the determination of apparent solubility, native FST and FHIC were added into 10 ml of PBS and stirred in an orbital shaker for 24 h (Madan et al., 2012). The solutions were centrifuged (10 000 rpm for 15 min), supernatant was collected and filtered through 0.22 μm filter. After appropriate dilution, FST absorbance was measured using UV-visible spectroscopy at 360 nm. The calibration curve was prepared in the range of 1–20 μg/ml. The observed regression equation was *Y* = 0.054*X* + 0.058 with *r*^2^=0.997.

## Characterization of FHIC solid complex

### FTIR spectroscopy

The interaction between FST (guest molecule) and HPβCD (host molecule) was studied with the aid of infrared spectroscopy (Perkin Elmer, Spectrum One, Waltham, MA). The spectra of FST, HPβCD, dried inclusion complex (FHIC) and physical mixture of FST and HPβCD (FHPM) were recorded by scanning between 4000 and 400 cm^−1^ with a resolution of 4 cm^−1^. The KBr pellets of samples were prepared by mixing 3 mg of sample in 100 mg of KBr.

### Differential scanning calorimetry (DSC)

A differential thermal scanning was carried out to observe the endothermic peaks of FST, FH PM and FHIC using DSC-Q100 (TA Instruments, New Castle, DE) and analyzed by Mettler Toledo STAR system software (Columbus, OH). An amount of 3.5 mg of the sample was introduced into the aluminum pan which was exposed to a temperature range of 50–300 °C/min at a heating rate of 10 °C/min. Nitrogen was purged at a flow rate of 50 ml/min.

### Powder X-ray diffractometry (PXRD)

An X-ray diffraction analysis was carried out on a D8 Advance, Bruker, Karlsruhe, Germany at 40 kV and 30 mA with CuK_α_ radiation to determine the crystal bond lattices of FST, FHPM and FHIC.

### Proton nuclear magnetic resonance (^1^H NMR) spectroscopy

The NMR spectra of FST, HPβCD and FHIC were observed using a BRUKER DPX 300 MHz spectrometer (Bruker, Karlsruhe, Germany). HPβCD and FHIC were solubilized in D_2_O while FST was dissolved in d_6_DMSO.

### Preparation and characterization of FHIC-PNP

PLGA nanoparticles were prepared using multiple emulsion technique (w/o/w) with slight modifications (Gidwani & Vyasa, 2016). FHIC (equivalent to 5 mg FST) were dissolved in 1 ml of pluronic F-68 (PF-68) solution (1% w/v) and transferred into 2 ml PLGA solution (25 mg/ml). The dispersion was sonicated for 2 min at 35% amplitude. This primary emulsion was again emulsified in 1%w/v TPGS solution (10 ml) under sonication for 3 min at 35% amplitude. Further, it was stirred magnetically for 3 h at 1000 rpm to evaporate the organic solvent.

FHIC loaded PNP were obtained by centrifugation at 10 000 rpm for 30 min at 10 °C and were washed with distilled water for three times. The obtained nanoparticles were freeze-dried and stored at 4 °C until further use.

### Assessment of particle diameter and zeta potential

The mean particle diameter, polydispersity index and zeta potential of FHIC-PNP and blank PNP were measured using a Zetasizer Nano ZS (Malvern Instruments, Malvern, UK). The measurement was performed at 25 °C after 1:100 dilutions of samples in distilled water. Each measurement was performed in triplicate and the average value was considered as particle diameter or zeta potential.

### Encapsulation efficiency

The amount of drug loaded in the nanoparticles was determined by indirect method. Nanoparticle dispersion was centrifuged using Centrisart tubes (Sartorius, Bohemia, NY) and supernatant was analyzed for FST content using UV-visible spectrophotometry at 360 nm. The EE was calculated as follows: EE (%) = {(*D*_T_ – *D*_S_)/*D*_T_} × 100, where *D*_T_=total FST added; *D*_S_=FST in the supernatant.

### Stability of FHIC-PNP in simulated GI fluids

FHIC-PNP were dispersed in simulated gastric fluid pH 1.2 (SGF) and simulated intestinal fluid pH 6.8 (SIF) and change in particle diameter, zeta potential and entrapment efficiency were observed.

### *In vitro* drug release studies

Dialysis bag method was used to carry out the *in vitro* drug release studies. FST or FHIC-PNP dispersion equivalent to 1 mg of FST, was introduced into the dialysis bag (12 000–14 000 molecular weight cut off, Sigma Aldrich, St. Louis, MO) and placed in 100 ml of phosphate buffer saline pH 7.4 (PBS) containing 0.1%v/v Tween 80 as the release media and kept for stirring at 37 °C at 100 rpm. Samples were withdrawn at different time intervals and replaced with equal volume of media to maintain the sink condition. The collected samples were analyzed for amount of FST content by UV-visible spectrophotometry at 360 nm.

### *In vitro* biological experiments

#### Cell viability assay

The cell viability assay (MTT assay) was performed in MCF-7 human breast adenocarcinoma cells. Cells were seeded at a density of 7 × 10^3^ in DMEM medium supplemented with 10% FBS in a 96-well plate and incubated for 24 h at 37 °C in a humidified 5% CO_2_. The cells were exposed to different concentrations of FST, FHIC and FHIC-PNP for 24 h in order to ascertain the concentration at which 50% of the cells are viable. A volume of 10 μl of MTT solution (5 mg/ml in PBS) was added to each well. The plate was incubated for 4 h and thereafter the contents in the wells were replaced with 100 μl DMSO and again incubated for 10 min. The optical density was measured using a microplate reader (BioTek Instruments, Synergy 4, Winooski, VT) at 570 nm. The cytotoxicity of blank PNP was also studied to determine the toxicity of the nanocarrier system.

#### Intracellular localization study

To study the intracellular localization of FHIC-PNP and FHIC, coumarin 6 loaded PLGA nanoparticles (C6-IC-PNP) and coumarin 6 containing inclusion complex (C6-IC) were prepared in the similar way by replacing FST with coumarin 6. MCF-7 cells (5 × 10^4^) were seeded in 24-well plate and incubated for 24 h. The media were replaced with 20 μg/ml of C6-IC and C6-IC-PNP and further incubated for 1 h, 2 h or 4 h at 37 °C. The cells were washed with ice cold PBS and were fixed with 4% paraformaldehyde in PBS for 30 min at room temperature. The cells were observed using a fluorescence microscope fixed with FITC filters (Nikon, Inc., Tokyo, Japan).

For quantitative cellular uptake studies, cells were lysed with 0.5%v/v Triton-X in PBS for 5 min. The cell lysate was collected and fluorescence intensity was measured using a microplate reader (BioTek Instruments, Synergy 4, Winooski, VT).

#### Qualitative analysis of apoptosis and nuclear morphology by Hoechst staining

MCF-7 cells (1 × 10^5^) were seeded in 12-well plate and treated with FST, FHIC or FHIC-PNP (equivalent to 20 μg/ml FST) for 24 h. The cells were subsequently washed with PBS and fixed with 4% formaldehyde. The cell nuclei were then stained with Hoechst 33258 (5 μg/ml) for 20 min and observed under fluorescence microscope (Nikon, Tokyo, Japan). Apoptotic cells were recognized by fragmented or condensed nuclei.

#### Quantitative analysis of apoptosis using flow cytometer

For the quantitative analysis of apoptosis, cells were treated with FST, FHIC or FHIC-PNP (equivalent to 20 μg/ml FST) for 24 h. Cells were trypsinized, centrifuged at 1200 rpm for 5 min, washed three times with ice-cold PBS and were re-suspended in 500 μl of binding buffer. Thereafter, 5 μl of Annexin V-FITC and 10 μl of PI were added and mixed for 15 min in the dark. The stained cells were analyzed using a flow cytometer (FACS Calibur, BD, San Jose, CA). Data analysis was performed using Cell-Quest software (Becton Dickinson, Franklin Lakes, NJ).

#### Reactive oxygen species (ROS) assay

MCF-7 cells (1 × 10^5^) were exposed to different formulations viz. FST, FHIC or FHIC-PNP (equivalent to 20 μg/ml FST) for 24 h. Subsequently 5 μM of 2′,7′-dichlorodihydrofluorescein diacetate (DCFDA) was added and cultured for additional 30 min. Cells were trypsinized with 0.5% v/v Triton-X and intracellular ROS was quantified as a function of fluorescence intensity, measured using a microplate reader (BioTek Instruments, Synergy 4, Winooski, VT).

### *In vivo* studies

#### Pharmacokinetic studies

The animal experiments were performed in accordance with the Committee for the Purpose of Control and Supervision of Experiments on Animals (CPCSEA) guidelines after approval from Institutional Animal Ethics Committee (IAEC) of the institution (Approval no. IICT/39/2016). Female C57BL6 mice weighing from 18 to 22 g were kept for overnight fasting before the start of the experiment and allowed free access of water *ad libitum*.

C57BL6 mice were divided into two groups containing 27 animals in each. Animals in group I received pure FST (as 1% gum acacia suspension) orally at a dose of 10 mg/kg body weight and the animals in group II received FHIC-PNP (FST equivalent to 10 mg/kg) orally. At predetermined time intervals, blood samples were collected from retro-orbital plexus into centrifuge tube containing EDTA. Samples were centrifuged at 4000 rpm for 15 min and supernatant (plasma) was collected and stored at −80 °C for further analysis by LC/MS.

### Bioanalytical method

#### Plasma samples processing

Plasma samples were extracted by solvent mixture composed of chloroform and acetonitrile (1:1). The mixture was kept in shaker for 10 min and the supernatant layer was transferred into another Eppendorf tubes. Evaporation of organic solvent was done on ScanVac speed vacuum concentrator and 100 μl of acetonitrile was added and vortexed. A volume of 2 μl aliquots of sample solution were injected into LC–MS/MS for analysis. The linearity of the method was evaluated by analyzing spiked calibration samples using six calibration standards over a calibration range of 0.5–800 ng/ml in mice plasma.

Analysis was carried out on U-HPLC instrument (Agilent Technologies, Santa Clara, CA) equipped with a quaternary pump, a degasser, a diode array detector, an auto sampler and a column compartment. Mass spectrometric analysis was carried out on an Agilent 6420 triple quadrupole (Agilent Technologies, Santa Clara, CA) equipped with electrospray ionization (ESI) source. The data acquisition was under the control of Mass Hunter software.

FST and baicalein, an internal standard (IS), were separated on Agilent Eclipse plus C18 (2.1 mm × 50 mm, 1.8 μm) at constant temperature of 40 °C. The initial mobile phase consisted of 10 mM of ammonium formate (pH 3.5) and acetonitrile with gradient elution at a flow rate of 0.4 ml/min. Elution was in a gradient, the acetonitrile was kept constant at 2% in 0.5 min, and then increased to 98% in 1.5 min and maintained at 98% for another 2 min, then decreased to 2% in 3.8 min and maintained for 2.2 min. The total run time of the analysis was 5 min. After each injection, the sample manager was cleaned by a needle wash process with methanol–water (70/30 v/v). The typical operating source condition for MS scan in negative ion ESI mode were optimized as follow: collision energy 33 eV, cell accelerator voltage 7 eV, fragmentor 135, dwell time 200 ms, gas temperature 280 °C, gas flow 10 l/min and nebulizer 50 psi. The MRM modes of ions with *m*/*z* 284.8 → 134.8 for FST and *m*/*z* 268.8 → 166.8 for IS (baicalein) were utilized for quantitative analysis.

#### Intestinal uptake study

Intestinal uptake study was performed to predict the absorption of FHIC-PNP through the intestine (Joshi et al., 2014). C57BL6 mice were fasted overnight and were orally administered with C6-FHIC and C6-FHIC-PNP. After 1 h of administration, mice were euthanized and the ileum region was dissected and washed thoroughly with PBS. The tissue samples with 10 μm of thickness were embedded in tissue freezing medium (Leica Biosystems, GmBH, Nussloch, Germany) and frozen at −30 °C. Each circular tissue section was prepared, mounted on glass slides and visualized under fluorescence microscope (Nikon, Inc., Tokyo, Japan, 6-coumarin: *λ*_ex_ 430, *λ*_em_ 433–485 nm; green fluorescence).

### Statistical analysis

All experiments were performed in triplicate (*n* = 3) and results are expressed as mean ± SD. The statistical analysis was done by one-way analysis of variance (ANOVA) with Dunnett’s multiple comparison test using GraphPad Prism software (version 6.0). Statistical difference between two groups was determined by Student’s *t*-test. A probability level of *p* < 0.05 was considered as significant.

## Results and discussion

### Preparation and characterization of FHIC

FST was complexed with HPβCD to improve its solubility in water. HPβCD is a truncated cone-shaped molecule with a hollow, tapered cavity that allows incorporation of small molecules. The exterior of cavity of HPβCD is highly hydrophilic due to presence of the bristling hydroxy groups, while the interior is slightly lipophilic. This unique property of HPβCD makes it suitable candidate for the development of inclusion complexes with hydrophobic molecules. Moreover, it can also form reversible and noncovalent inclusion complexes with compounds that geometrically fit inside the cavity. FHIC formation is expected through non-covalent interaction, i.e. hydrogen bonding with hydroxyl groups present on both FST and HPβCD. Further, for the determination of stoichiometry ratio of FST and HPβCD in FHIC, FST was incorporated into the PBS containing HPβCD ranging from 2 to 16 mM. Supplementary Figure S1 shows the phase solubility diagram of FST in FHIC at 37 °C. The solubility of FST was increased linearly with increase in HPβCD and presented an *A*_L_ type of curve according to Higuchi and Connor equation. The slope of the linear correlation suggested a 1:1 mM complex formation between FST and HPβCD in the solution phase with an apparent stability constant (*K*_C_) of 1296.03 M^−1^. The apparent solubility of FST was increased from 5.25 μg/ml to 850 μg/ml after inclusion complex formation.

An infrared spectroscopic study was performed to confirm the complexation of FST with HPβCD. The FTIR spectrum of FST revealed absorption bands appearing at 1616 cm^−1^ (C=O stretching), 1571 cm^−1^ (C–C stretching), 1504 cm^−1^ (C–O stretching) and 1273 cm^−1^ (C–O–H bending). HPβCD shows prominent peaks at 3387 cm^−1^ (O–H), 2930 cm^−1^ (C–H) and 1655 cm^−1^ (H–O–H bending) (Jeong et al., 2013). The physical mixture of FST with HPβCD showed additive effect of FTIR spectra of FST and HPβCD indicating a simple superimposition of spectra of individual component. However the inclusion complex spectra of FST-HPβCD showed diminished bands at 1618 cm^−1^ (C=O stretching), 1570 cm^−1^ (C–C stretching) and no peak at 1280 cm^−1^ (C–O–H bending) indicating incorporation of FST into the cavity of HPβCD (Supplementary Figure S2(a)).

The solid state interaction between FST and HPβCD was determined by DSC analysis. FST exhibited a sharp endothermic peak at 344.58 °C corresponding to its melting point (Liu et al., 2014) (Supplementary Figure S2(b)) while pure HPβCD showed a broad endothermic peak in between 50 and 80 °C due to the release of water molecules (de Araújo et al., 2008). The physical mixture of FST-HPβCD (FHPM) showed a broad endotherm for HPβCD at around 80 °C and a slight shift in the peak of FST indicating no or a very less interaction. In contrast, the endothermic peak of FST was not observed in FHIC thereby indicating a complex formation of FST with HPβCD.

The X-ray diffraction patterns of FST, its physical mixture with HPβCD and FHIC complex are shown in Supplementary Figure S2(c). The diffraction pattern of FST exhibited characteristic peaks at 12.5*θ*, 14.2*θ*, 16*θ*, 17.8*θ*, 24.2*θ* and 28.9*θ*, indicating crystallinity whereas absence of sharp peaks in the diffraction pattern of HPβCD suggested amorphous nature of native HPβCD. The diffraction pattern of the physical mixture was simply a superimposition of the individual spectra of FST and HPβCD indicating lack of interaction between both the molecules. Comparatively the diffraction pattern of inclusion complex (FHIC) showed overlapping with the spectra of HPβCD with absence of crystalline peaks of FST. These results indicated the formation of new complex compounds which existed as amorphous state.

FHIC was also characterized by ^1^H NMR analysis that provides a direct evidence of complex formation between FST and HPβCD (Supplementary Figure S2 (d)). FHIC showed the characteristic peaks of protons of both HPβCD and FST. NMR spectra of HPβCD showed the characteristic peaks of H-1 protons at *δ*5.079 and *δ*5.231, H-3 protons at *δ*3.891 and H-6 protons at *δ*3.745. After complexation with FST (FHIC), these peaks appeared at *δ*5.079, *δ*5.237, *δ*3.878 and *δ*3.731, respectively. The results suggested that H-3 (▵*δ* 0.013) and H-5 protons (▵*δ* 0.014) showed higher shift as compared to H-1 protons (▵*δ* 0.08). It can be explained on the basis of the orientation of protons. H-3 and H-5 protons are present in the inner surface of HPβCD and interact more with FST molecules present in its cavity than the H-1 protons which are present on the outer surface of HPβCD cavity (Zhang et al., 2015). Similarly, the aromatic protons of native FST were observed in the range of *δ*6.932–*δ*6.953 which were shifted to the *δ*6.979–*δ*6.994 in FHIC.

### Preparation of FHIC-PNP

FHIC-PNP were prepared by multiple emulsification technique (w/o/w). In the present study, PF68 and TPGS were used as surfactants for inner and outer aqueous phase, respectively. Ethyl acetate was chosen as solvent because of its lower toxicity and belongs to class III solvents classification system (USP, Organic volatile impurities, 2007). FHIC-PNP formulation was optimized (data not shown) to keep the particle size below 200 nm that facilitates the uptake of nanoparticles in the intestinal mucosa (Jiang et al., 2013). The optimized FHIC-PNP had the mean particle diameter of 87.27 ± 0.10 nm. The physicochemical parameters of PNP (with or without inclusion complex) are shown in Supplementary Table S1. The mean zeta potential of FHIC-PNP was −8.71 ± 0.03 mV. The nanoparticles with negative zeta potential are usually readily taken up by Peyer’s patches present in intestine and are translocated into the blood circulation in comparison to nanoparticles bearing positive charge (Joshi et al., 2014). With regard to the drug EE, it was observed that FST (as FHIC) was encapsulated more (79% versus 47.3%) in nanoparticles than pure FST. These results substantially fulfilled one of the major objectives of the present study. The percentage yield of FHIC-PNP was observed to be 96.32%.

### Stability studies in simulated biological fluids

After oral administration, nanoparticles need to pass through the stomach with highly acidic pH before reaching its absorption site in intestine. Hence, the stability of FHIC-PNP was studied in simulated SGF and SIF. The changes in various physicochemical parameters of FHIC-PNP after exposing to SGF and SIF are shown in Supplementary Table S2. About 4.1% and 11.6% of the FST were released after 2 h incubation in SGF and after 6 h in SIF, respectively. The insignificant change in particle size and zeta potential values of nanoparticles indicated the high physical stability of the FHIC-PNP in both the medium.

### *In vitro* drug release profile

**Figure 1.**
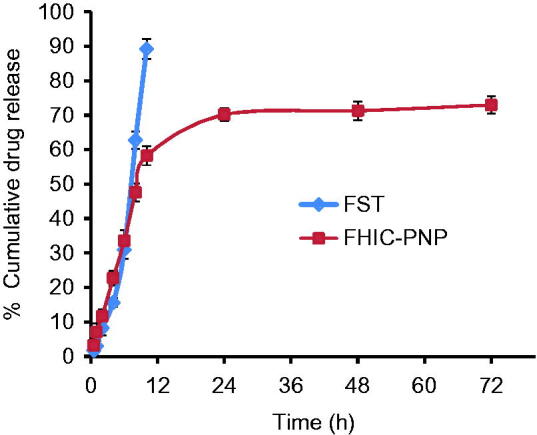
*In vitro* drug release profile of fisetin (FST) and FST-HPβCD complex loaded PLGA nanoparticles (FHIC-PNP) in phosphate buffer saline pH 7.4 (mean ± SD, *n* = 3).

The *in vitro* release behaviors of FST from FST solution and FHIC-PNP were compared (Figure 1). It was observed that about 93% of FST from FST solution was released within 8 h while only 43% was released from FHIC-PNP after same period of time. The sustained release of FST from FHIC-PNP was continued up to 72 h. The biphasic pattern of drug release from PNP is well known and is attributed to initial burst release of drug from the periphery of the nanoparticles followed by slow diffusion of the drug from inside the polymer matrix (Cheng et al., 2007; Kulhari et al., 2014; Zhang et al., 2016).

### *In vitro* anticancer activity of FHIC-PNP

**Figure 2.**
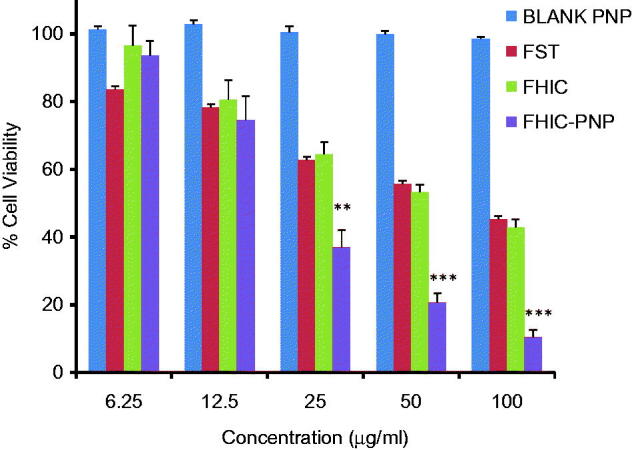
Concentration-dependent cytotoxicity of blank PLGA nanoparticles (blank PNP), fisetin (FST), FST-HPβCD complex (FHIC) and FHIC loaded PLGA nanoparticles (FHIC-PNP) against MCF-7 human breast cancer cells after 24 h of treatment. *Represents comparison between FHIC-PNP and FST. Statistical analysis: **p < 0.01, ***p < 0.001.

The *in vitro* cytotoxicity assays of FST, FHIC and FHIC-PNP were evaluated in MCF-7 cell line and IC_50_ values were compared. Figure 2 shows the anti-proliferative activity of three compounds to MCF-7 cells after 24 h of incubation which followed the order of FHIC-PNP (22.09 ± 1.3 μg/ml)>FHIC (68.11 ± 1.52 μg/ml)≥FST (77.83 ± 2.12 μg/ml) (Table S2). FHIC-PNP showed higher cytotoxicity as compared to FHIC and FST (*p* < 0.001) but an insignificant difference was observed between FHIC and FST. The higher cytotoxicity of FHIC-PNP could be attributed to enhanced intracellular delivery of FST by FHIC-PNP through endocytic internalization into the cells. Cells treated with blank nanoparticles showed more than 98% of cell viability indicating the biocompatibility of drug delivery system.

### Intracellular localization study

**Figure 3.**
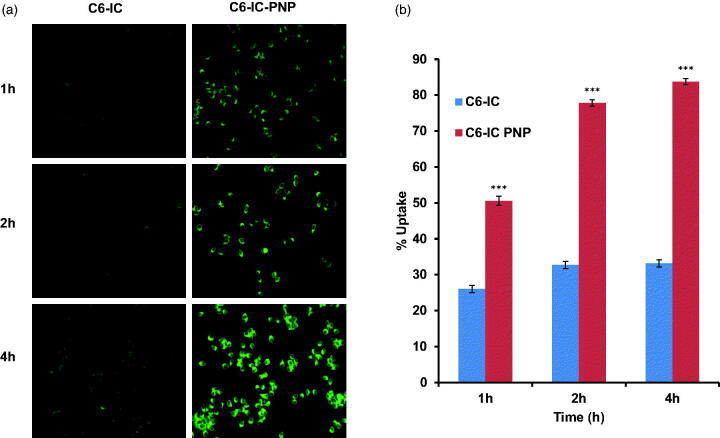
(a) Cellular uptake studies: fluorescent microscopic images of MCF-7 human breast cancer cells incubated with coumarin 6 complexed with HPβCD (C6-IC) and C6-IC loaded into PLGA nanoparticles (C6-IC-PNP) at different time points. (b) The quantitative cellular uptake of C6-IC and C6-IC-PNP (mean ± SD, *n* = 3). Statistical analysis: ***p < 0.0001 represents uptake of C6-IC-PNP versus C6-IC.

To confirm the internalization efficacy, C6-IC and C6-IC-PNP were used for the cellular uptake studies in MCF-7 cell lines. The uptake of both the formulations was time-dependent and florescence intensity was increased with the increase in time up to 4 h (Figure 3a). From Figure 3(b), it can also be observed that the uptake of C6-PNP was more as compared to C6-IC. Cells treated with C6-PNP showed higher fluorescence intensity at all the time points than C6-IC. There is ∼2.5 fold increase in transport of nanoparticles inside the cells after 4 h incubation as compared to C6-IC indicating that the uptake of the flavonoid into the cancer is mostly due to the nanoparticles. This might be due to the smaller size of the PNP which might have contributed to higher internalization into the cells thus exhibiting higher fluorescence.

Quantitative cellular uptake studies further confirmed that the cellular uptake efficiency of C6-IC-PNP was significantly (*p* < 0.0001) increased with incubation time from 1 h to 4 h, however there was no significant difference in the uptake of the C6-IC with time till 4 h. There is 1.6 fold increase in uptake of C6-IC-PNP after 4 h as compared to 1 h time interval.

### FST-PNP mediated apoptosis induction and ROS generation in MCF-7 cells

**Figure 4.**
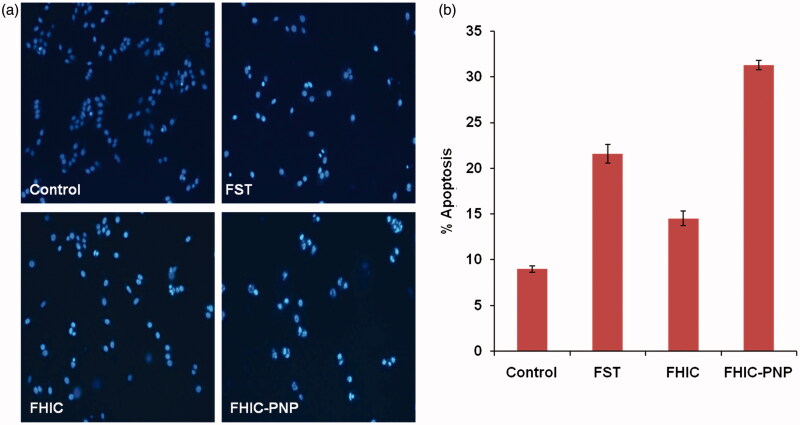
(a and b) Induction of apoptosis in MCF-7 human breast cancer cells after 24 h incubation with different samples, equivalent to 20 μg/ml fisetin. Cell nuclei are stained with Hoechst 33258.

FST causes apoptosis by activation of caspase-7, -8 and -9, and PARP cleavage in MCF-7 cells (Yang et al., 2011). To assess the presence of apoptotic cell bodies on treatment with various FST formulations, qualitative apoptosis assay was performed using Hoechst 33258 dye. The nuclei of control group showed homogenous blue fluorescence with round cell nuclei and absence of any fragmentation. However, MCF-7 cell lines treated with FST, FHIC and FHIC-PNP showed presence of fragmented nuclei distributed as apoptotic cell bodies after treatment for 24 h are shown in Figure 4(a). The presence of ruptured nuclei was markedly greater for FHIC-PNP compared to pure FST and FHIC.

**Figure 5.**
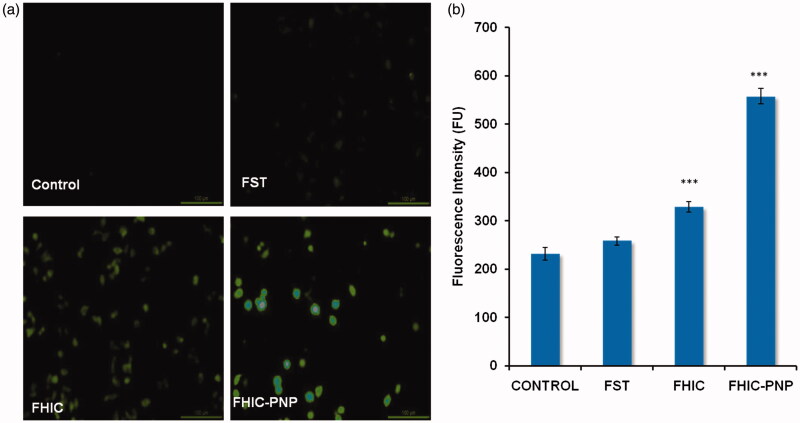
(a and b) Fluorescent microscopic images of MCF-7 cell line for reactive oxygen species assay (ROS) at equivalent FST concentration (20 μg/ml). Quantitative measurement of the fluorescent intensity in MCF-7 cells treated with FST, FHIC and FHIC-PNP at equivalent FST concentration (20 μg/ml). Data represented as mean ± SD (*n* = 3). Statistical analysis: ***p < 0.001, control versus FST, FHIC, FHIC-PNPs.

The apoptosis induced by FST, FHIC and FHIC-PNP was determined quantitatively using flow cytometry and was compared. The number of live cells was decreased from 89.8% to 59.6% in the cells-treated with FHIC-PNP compared to control cells. FHIC-PNP showed higher apoptosis induction (30.8%) than pure FST (21.8%) and FHIC (14.5%) as shown in Figure 4(b). FST also induce apoptosis through ROS generation in cancer cells (Jang et al., 2012). FST-induced ROS generation in MCF-7 cells was determined after incubation of cells with FST, FHIC or FHIC-PNP. As shown in Figure 5(a), there is complete absence of fluorescence in control group as indicating no or very less ROS generation. The fluorescence intensity of FHIC-PNP treated cells was observed to be higher significantly (*p* < 0.001) in comparison to FST and FHIC as demonstrated in Figure 5(b). Increase in the fluorescence intensity indicates higher ROS generation and increased oxidative stress thereby causing cell death.

### Pharmacokinetic studies

**Figure 6.**
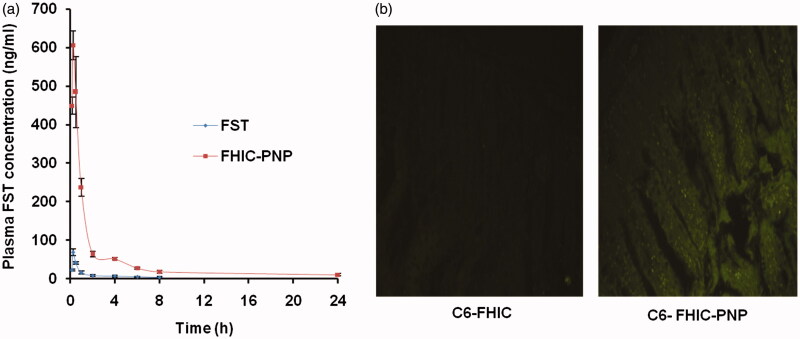
(a) Mean plasma concentration-time profiles of fisetin (FST) after oral administration as FST suspension and FST-HPβCD complex loaded PLGA nanoparticles (FHIC-PNP) in C57BL6 mice at a dose of 10 mg/kg body weight (mean ± SD, *n* = 3). (b) Fluorescent microscopic images of uptake of coumarin 6 in mice intestine after oral administration as C6-FHIC and C6-FHIC-PNP.

Figure 6(a) shows the plasma FST concentration–time profile and Table 1 summarizes the pharmacokinetic parameters of FST and FHIC-PNP oral administration at dose equivalent to 10 mg/kg of FST. The *C*_max_ value of FHIC-PNP (610.33 ng/ml) was significantly (*p* < 0.01) higher than pure FST (69.34 ng/ml). The lower *C*_max_ value of FST contributes to lower bioavailability. Nanoparticle mediated drug delivery through the encapsulation of FHIC into PLGA NPs considerably altered the pharmacokinetic profile. The 8.8 fold increase in *C*_max_ of FST as FHIC-PNP could be attributed to enhanced and rapid absorption of nanoparticles through M-cells of the Peyer patches in the intestine. This enhanced absorption further leads to increase in AUC of FHIC-PNP which represents the extent of total FST absorbed. There was an insignificant difference (*p* > 0.05) in the *t*_max_ of FST and FST-PNP. A similar kind of results was observed previously by Bothiraja et al., after extravascular (intraperitoneal) administration of pure FST and FST nanochelates (Bothiraja et al., 2014). Furthermore, the area under curve (AUC_0–_*_t_*) for FST-NP demonstrated 15.7 times increase which is significantly higher (*p* < 0.01) signifying higher oral bioavailability as compared to FST.

**Table 1.** Pharmacokinetic parameters of fisetin (FST) after oral administration as FST suspension and FST-HPβCD complex loaded PLGA nanoparticles (FHIC-PNP) in C57BL6 mice at a dose of 10 mg/kg body weight (mean ± SD, *n* = 3).

Pharmacokinetic parameters	FST suspension	FHIC-PNP
*C*_max_ (ng/ml)	69.34 ± 4.16	610.33 ± 5.31^b^
*t*_max_ (h)	0.25 ± 1.2	0.33 ± 0.14
AUC_0–_*_t_* (ng/ml/h)	80.39 ± 3.61	1259.48 ± 5.15^b^
AUC_0–∞_ (ng/ml/h)	100.13 ± 2.91	1340.05 ± 4.91^b^
*t*_1/2_ (h)	3.26 ± 0.90	5.86 ± 0.42^a^
Cl (ml/h)	2022.59 ± 0.02	158.13 ± 0.01^b^
MRT (h)	3.82 ± 2.15	6.38 ± 3.73^a^

*C*_max_: maximum (peak) concentration of fisetin in blood plasma; *t*_max_: observed time after fisetin administration at which peak plasma concentration occurs; AUC_0–_*_t_*: total area under the plasma fisetin concentration–time curve (from time zero to time t); AUC_0–∞_: total area under the plasma fisetin concentration–time curve (from time zero to infinity); *t*_1/2_: half-life of fisetin; Cl: systemic clearance; MRT: mean residence time of fisetin. Statistical analysis: a represents *p* < 0.05 and b represents *p* < 0.01.

## *Ex vivo* intestinal uptake study

Figure 6(b) shows penetration of C6-PNP in the intestinal mucosa of rat as shown by the green fluorescence but there is negligible fluorescence in C6-FHIC-PNP which confirmed the transport of the nanoparticles through the intestinal region of the GI tract.

## Conclusion

FST was complexed with HPβCD and thereafter successfully incorporated into PLGA NP for high drug loading, improved anticancer activity and oral bioavailability. FHIC-PNP showed ∼79% encapsulation of FST as inclusion complex with HPβCD which was otherwise only 47% with native FST. The developed nanoformulations showed 3.9 times higher toxicity compared to pure FST against MCF-7 human breast cancer cell lines. FST-PNP also enhanced the FST-induced apoptosis and ROS generation in cancer cells. *In vivo* studies in C57BL6 mice revealed that incorporation of FHIC in FHIC-PNP significantly improved the pharmacokinetics and oral bioavailability of FST. Therefore, the present strategy can be further explored for the delivery of hydrophobic therapeutic agents.

## Supplementary Material

Supplementary_File.doc

## Notes

**Supplementary material available online**

## References

[CIT0001] Adhami VM, Syed DN, Khan N, et al. (2012). Dietary flavonoid fisetin: a novel dual inhibitor of PI3K/Akt and mTOR for prostate cancer management. Biochem Pharmacol 84:1277–81 22842629 10.1016/j.bcp.2012.07.012 PMC3813433

[CIT0002] Bhat TA, Nambiar D, Pal A, et al. (2012). Fisetin inhibits various attributes of angiogenesis in vitro and in vivo – implications for angioprevention. Carcinogenesis 33:385–93 22139440 10.1093/carcin/bgr282

[CIT0003] Bothiraja C, Yojana BD, Pawar AP, et al. (2014). Fisetin-loaded nanocochleates: formulation, characterisation, in vitro anticancer testing, bioavailability and biodistribution study. Expert Opin Drug Deliv 11:17–29 24294925 10.1517/17425247.2013.860131

[CIT0004] Cheng J, Teply BA, Sherifi I, et al. (2007). Formulation of functionalized PLGA-PEG nanoparticles for in vivo targeted drug delivery. Biomaterials 28:869–76 17055572 10.1016/j.biomaterials.2006.09.047 PMC2925222

[CIT0005] Chou RH, Hsieh SC, Yu YL, et al. (2013). Fisetin inhibits migration and invasion of human cervical cancer cells by down-regulating urokinase plasminogen activator expression through suppressing the p38 MAPK-dependent NF-κB signaling pathway. PLoS One 8:e71983 23940799 10.1371/journal.pone.0071983 PMC3733924

[CIT0006] de Araújo MV, Vieira EK, Silva LG, et al (2008). Sulfadiazine/hydroxypropyl-beta-cyclodextrin host-guest system: characterization, phase-solubility and molecular modeling. Bioorg Med Chem 16:5788–94 18434167 10.1016/j.bmc.2008.03.057

[CIT0007] Dua F, Menga H, Xua K, et al. (2014). CPT loaded nanoparticles based on beta-cyclodextrin-grafted poly(ethylene glycol)/poly(l-glutamic acid) diblock copolymer and their inclusion complexes with CPT. Colloids Surf B: Biointerfaces 113:230–6 24096159 10.1016/j.colsurfb.2013.09.015

[CIT0008] Gidwani B, Vyasa A. (2016). Formulation, characterization and evaluation of cyclodextrin-complexed bendamustine-encapsulated PLGA nanospheres for sustained delivery in cancer treatment. Pharm Dev Technol 21:161–71 25391288 10.3109/10837450.2014.979945

[CIT0009] Hu L, Zhang H, Song W, et al (2012). Investigation of inclusion complex of cilnidipine with hydroxypropyl-β-cyclodextrin. Carbohydr Polym 90:1719–24 22944438 10.1016/j.carbpol.2012.07.057

[CIT0010] Jang KY, Jeong SJ, Kim SH, et al. (2012). Activation of reactive oxygen species/AMP activated protein kinase signaling mediates fisetin-induced apoptosis in multiple myeloma U266 cells. Cancer Lett 319:197–202 22261340 10.1016/j.canlet.2012.01.008

[CIT0011] Jeong D, Choi JM, Choi Y, et al. (2013). Complexation of fisetin with novel cyclosophoroase dimer to improve solubility and bioavailability. Carbohydr Polym 97:196–202 23769537 10.1016/j.carbpol.2013.04.066

[CIT0012] Jiang L, Li X, Liu L, et al. (2013). Thiolated chitosan-modified PLA-PCL-TPGS nanoparticles for oral chemotherapy of lung cancer. Nanoscale Res Lett 8:66 23394588 10.1186/1556-276X-8-66 PMC3598981

[CIT0013] Joshi G, Kumar A, Sawant K. (2014). Enhanced bioavailability and intestinal uptake of Gemcitabine HCl loaded PLGA nanoparticles after oral delivery. Eur J Pharm Sci 60:80–9 24810394 10.1016/j.ejps.2014.04.014

[CIT0014] Kang KA, Piao MJ, Hyun JW. (2015). Fisetin induces apoptosis in human nonsmall lung cancer cells via a mitochondria-mediated pathway. In Vitro Cell Dev Biol Anim 51:300–9 25381036 10.1007/s11626-014-9830-6

[CIT0015] Kulhari H, Pooja D, Shrivastava S, et al. (2014). Peptide conjugated polymeric nanoparticles as a carrier for targeted delivery of docetaxel. Colloids Surf B Biointerfaces 117:166–73 24632389 10.1016/j.colsurfb.2014.02.026

[CIT0016] Kulhari H, Pooja D, Singh MK, et al. (2015). Bombesin-conjugated nanoparticles improve the cytotoxic efficacy of docetaxel against gastrin-releasing but androgen-independent prostate cancer. Nanomedicine UK 10:2847–59 10.2217/nnm.15.107 26377157

[CIT0017] Liu M, Cao W, Sun Y, et al. (2014). Preparation, characterization and in vivo evaluation of formulation of repaglinide with hydroxypropyl-β-cyclodextrin. Int J Pharm 477:159–66 25455768 10.1016/j.ijpharm.2014.10.038

[CIT0018] Madan J, Baruah B, Nagaraju M, et al. (2012). Molecular cycloencapsulation augments solubility and improves therapeutic index of brominated noscapine in prostate cancer cells. Mol Pharm 9:1470–80 22540277 10.1021/mp300063v PMC3428378

[CIT0019] Madan J, Gundala S, Baruah B, et al. (2014). Cyclodextrin complexes of reduced bromonoscapine in guar gum microspheres enhance colonic drug delivery. Mol Pharm 11:4339–49 25350222 10.1021/mp500408n PMC4255741

[CIT0020] Maurya BK, Trigun SK. (2016). Fisetin modulates antioxidant enzymes and inflammatory factors to inhibit aflatoxin-B1 induced hepatocellular carcinoma in rats. Oxid Med Cell Long 2016:1–9 10.1155/2016/1972793 PMC4670673 26682000

[CIT0021] Mudshinge SR, Deore AB, Patil S, et al. (2011). Nanoparticles: emerging carriers for drug delivery. Saudi Pharm J. 19:129–41 23960751 10.1016/j.jsps.2011.04.001 PMC3744999

[CIT0022] Noh EM, Park YJ, Kim JM, et al. (2015). Fisetin regulates TPA-induced breast cell invasion by suppressing matrix metalloproteinase-9 activation via the PKC/ROS/MAPK pathways. Eur J Pharmacol 764:79–86 26101063 10.1016/j.ejphar.2015.06.038

[CIT0023] Pooja D, Babu Bikkina DJ, Kulhari H, et al. (2014). Fabrication, characterization and bioevaluation of silibinin loaded chitosan nanoparticles. Int J Biol Macromol 69:267–73 24863917 10.1016/j.ijbiomac.2014.05.035

[CIT0024] Qiu N, Cheng X, Wang G, et al. (2014). Inclusion complex of barbigerone with hydroxypropyl-β-cyclodextrin: preparation and in vitro evaluation. Carbohydr Polym 101:623–30 24299819 10.1016/j.carbpol.2013.09.035

[CIT0025] Sah H, Thoma LA, Desu HR, et al. (2013). Concepts and practices used to develop functional PLGA-based nanoparticulate systems. Int J Nanomed 8:747–65 10.2147/IJN.S40579 PMC3582541 23459088

[CIT0026] Seguin J, Brullé L, Boyer R, et al. (2013). Liposomal encapsulation of the natural flavonoid fisetin improves bioavailability and antitumor efficacy. Int J Pharm 444:146–54 23380621 10.1016/j.ijpharm.2013.01.050

[CIT0027] Suh Y, Afaq FJJ, Mukhtar H. (2009). A plant flavonoid fisetin induces apoptosis in colon cancer cells by inhibition of COX2 and Wnt/EGFR/NF-kappaB-signaling pathways. Carcinogenesis 30:300–7 19037088 10.1093/carcin/bgn269 PMC2722149

[CIT0028] Tripathi R, Samadder T, Gupta S, et al. (2011). Anticancer activity of a combination of cisplatin and fisetin in embryonal carcinoma cells and xenograft tumors. Mol Cancer Ther 10:255–68 21216935 10.1158/1535-7163.MCT-10-0606

[CIT0029] U.S. Pharmacopeial Convention. (2007). Organic volatile impurities/residual solvents. General Chapter 467. Available at: http://www.usp.org/usp-nf/official-text/accelerated-revision-process/accelerated-revision-history/general-chapter-organic-volatile

[CIT0031] Yang PM, Tseng HH, Peng CW, et al. (2011). Dietary flavonoid fisetin targets caspase-3-deficient human breast cancer MCF-7 cells by induction of caspase-7-associated apoptosis and inhibition of autophagy. Int J Oncol 40:469–78 21922137 10.3892/ijo.2011.1203

[CIT0032] Yi C, Zhang Y, Yu Z, et al. (2014). Melatonin enhances the anti-tumor effect of fisetin by inhibiting COX-2/iNOS and NF-κB/p300 signaling pathways. PLoS One 9:e99943 25000190 10.1371/journal.pone.0099943 PMC4085069

[CIT0033] Zhang H, Firempong CK, Wang Y, et al. (2016). Ergosterol-loaded poly(lactide-co-glycolide) nanoparticles with enhanced in vitro antitumor activity and oral bioavailability. Acta Pharmacol Sin 37:834–44 27133301 10.1038/aps.2016.37 PMC4954769

[CIT0034] Zhang JQ, Jiang KM, An K, et al. (2015). Novel water-soluble fisetin/cyclodextrins inclusion complexes: preparation, characterization, molecular docking and bioavailability. Carbohydr Res 418:20–8 26531135 10.1016/j.carres.2015.09.013

[CIT0035] Zhao L, Feng SS. (2010). Enhanced oral bioavailability of paclitaxel formulated in vitamin E-TPGS emulsified nanoparticles of biodegradable polymers: in vitro and in vivo studies. J Pharm Sci 99:3552–60 20564384 10.1002/jps.22113

