# Supplementary material for: Enhanced oral bioavailability and anticancer efficacy of fisetin by encapsulating as inclusion complex with HPβCD in polymeric nanoparticles
Source: Drug Deliv. 2017 Feb 3;24(1):224–32. doi: 10.1080/10717544.2016.1245366 (PMC8241160; doi:10.1080/10717544.2016.1245366)
Supplement: Supplementary_File.doc [file IDRD_A_1245366_SM8060.doc]

**SUPPLEMENTARY MATERIAL**

**Enhanced oral bioavailability and anticancer efficacy of Fisetin by encapsulating as inclusion complex with HPβCD in polymeric nanoparticles**

Amrita Kadari^1^, Sagarika Gudem^1^, Hitesh Kulhari^1^, Murali Mohan Bhandi^2^, Roshan M. Borkar^2^, Venkata Ramana Murthy Kolapalli ^3^, Ramakrishna Sistla^1*^

^1^Medicinal Chemistry & Pharmacology Division, CSIR-Indian Institute of Chemical Technology, Hyderabad, Telangana, 500007, India

^2^National Centre for Mass Spectrometry, Indian Institute of Chemical Technology, Tarnaka, Hyderabad, India, 500007

^3^Department of Pharmaceutical Sciences, A.U. College of Pharmaceutical Sciences, Andhra University, Visakhapatnam 530003, India

***** Medicinal Chemistry & Pharmacology Division, CSIR-Indian Institute of Chemical Technology, Hyderabad, Telangana, 500007, India

*Email:* sistla@iict.res.in

**Supplemental file legends**

**Figure S1.** Phase solubility analysis of fisetin (FST) with hydroxypropyl beta cyclodextrin (HPβCD) in phosphate buffer pH7.4

**Figure S2 (a).** FTIR spectra of fisetin (FST), hydroxypropyl beta cyclodextrin (HPβCD), physical mixture of FST and HPβCD (FHPM), and inclusion complex of FST and HPβCD (FHIC)

**Figure S2 (b).** Differential scanning calorimetry of a) hydroxypropyl beta cyclodextrin b) fisetin c) physical mixture of FST and HPβCD d) inclusion complex of FST and HPβCD

**Figure S2 (c ).** PXRD pattern of fisetin (FST), hydroxypropyl beta cyclodextrin (HPβCD), physical mixture of FST and HPβCD (F-PM), and inclusion complex of FST and HPβCD (FHIC).

**Figure S2 (d)**. ^1^HNMR spectra of fisetin (FST) in DMSO, hydroxypropyl beta cyclodextrin (HPβCD) and inclusion complex of FST and HPβCD (FHIC) in D2O

**
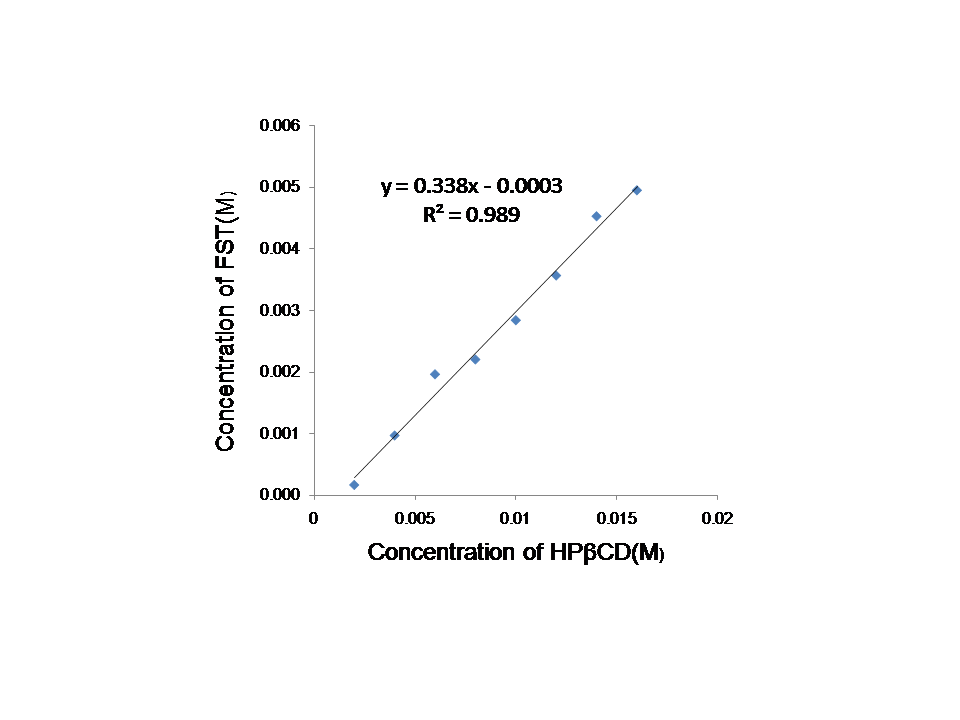
**

**Figure S1.**

**
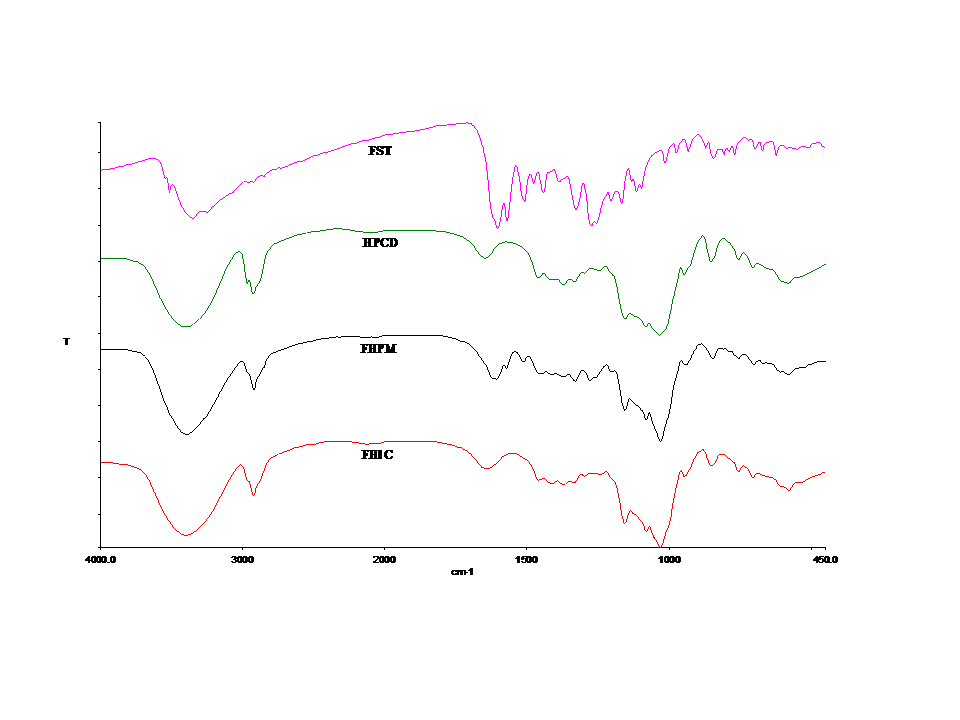
**

**Figure S2 (a).**

**
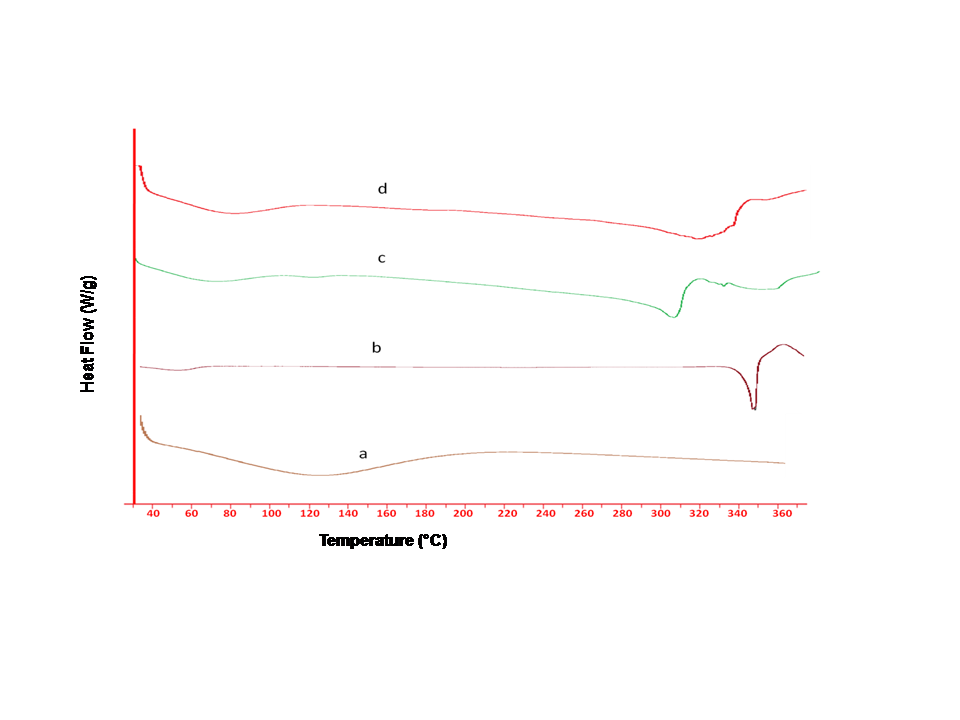
**

**Figure S2 (b).**

**
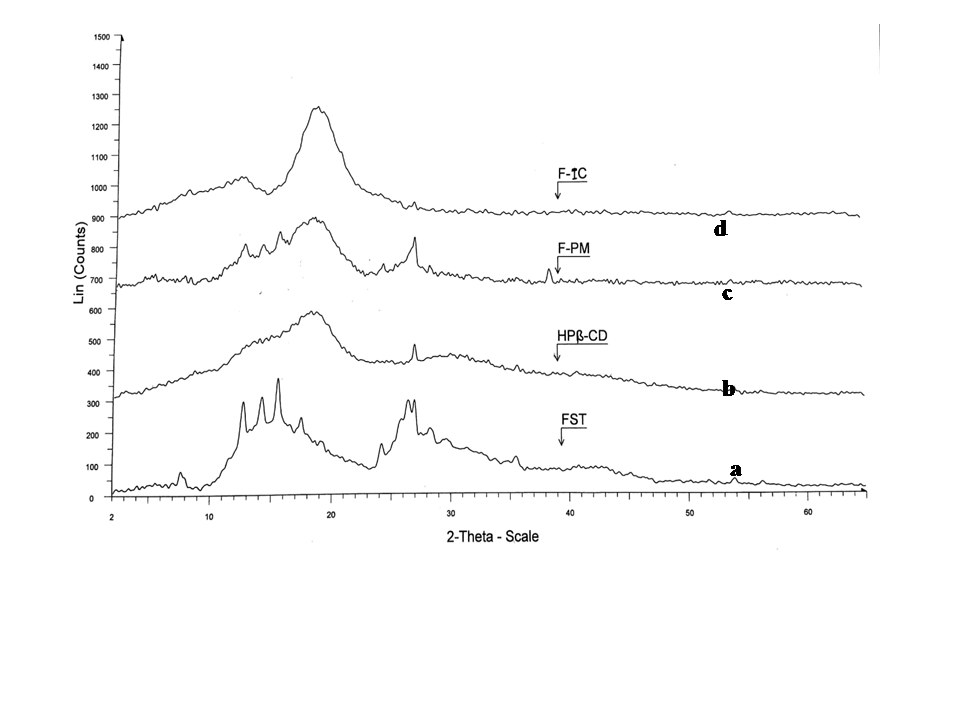
**

**Figure S2 (c).**

**
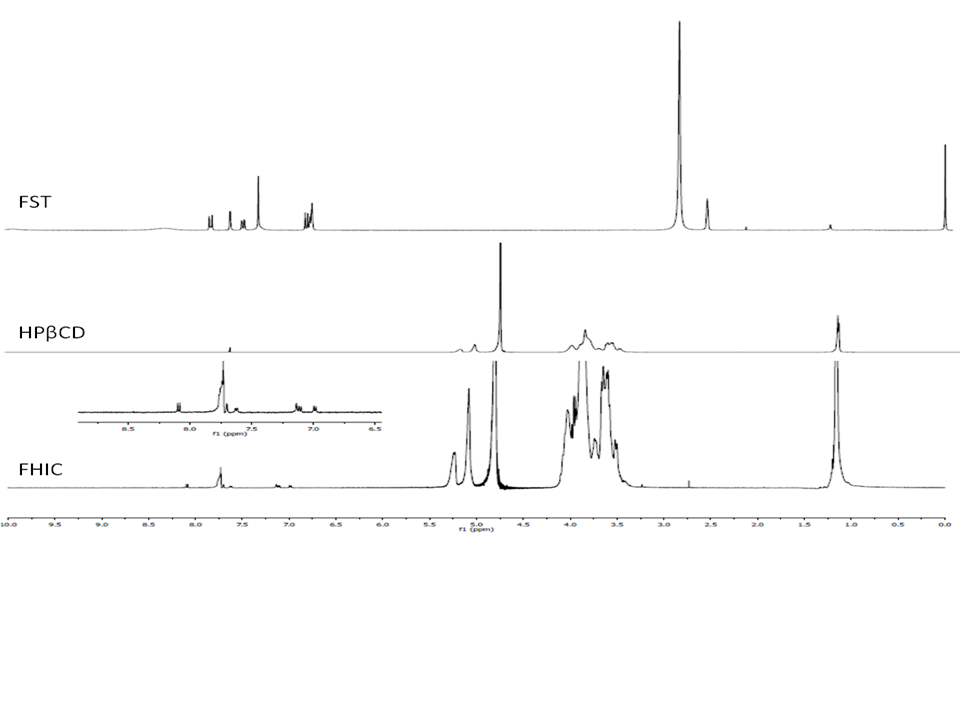
**

**Figure S2 (d)**.

**Table S1.** Particle diameter (PD), polydispersity index (PDI), zeta potential (ZP) and entrapment efficiency (EE) of blank PLGA nanoparticles (B-PNP) and FST and HPβCD inclusion complex loaded PLGA nanoparticles (FHIC-PNP). (Mean ± SD, n=6)

| **Parameters** | **B-PNP** | **FHIC-PNP** |
| --- | --- | --- |
| PD (nm) | 77.26 ± 0.94 | 87.27 ± 0.10 |
| PDI | 0.26 ± 0.05 | 0.25 ± 0.01 |
| ZP (mV) | -8.08 ± 0.10 | -8.71 ± 0.03 |
| EE (%) | - | 78.8 ± 0.55 |

**Table S2:** Stability studies of fisetin and HPβCD inclusion complex loaded PLGA nanoparticles in (A) Simulated gastric fluid pH 1.2 and (B) Simulated intestinal fluid pH 6.8

**A.**

| **Time (h)** | **Particle diameter (nm)** | **Zeta potential (mV)** | **EE (%)** |
| --- | --- | --- | --- |
| **1** | 98.24±1.3 | 7.13±0.041 | 79.6±1.46 |
| **2** | 99.69±0.33 | 7.18±0.065 | 79.5±0.18 |
| **4** | 102.7±0.11 | 8.39±0.23 | 78.1±0.01 |
| **6** | 100.7±1.19 | 7.4±0.21 | 78.2±0.02 |
| **8** | 105.42±0.38 | 7.22±0.015 | 78.9±0.04 |
| **24** | 107.48±0.17 | 7.57±0.16 | 78.55±0.17 |

**B.**

| **Time (h)** | **Particle diameter (nm)** | **Zeta potential (mV)** | **EE (%)** |
| --- | --- | --- | --- |
| **1** | 97.21 ± 0.82 | 6.67±0.005 | 80.49±0.42 |
| **2** | 100.45± 0.17 | 7.14±0.003 | 73.3±5.42 |
| **4** | 101.65± 0.04 | 2.17±7.2 | 78.4±0.29 |
| **6** | 106.25± 0.78 | 7.15±0.025 | 80.4±1.28 |
| **8** | 106.65± 0.40 | 7.47±0.45 | 81.3±0.11 |
| **24** | 111.54± 0.20 | 7.4±0.07 | 81.41±0.20 |

**Table S3:**  IC50 dose of fisetin (FST), FST-HPβCD complex (FHIC) and FHIC loaded PLGA nanoparticles (FHIC-PNP) against MCF-7 human breast cancer cells after 24 h of treatment. (Mean ± SD, n=3)

| **Formulations** | **IC_50_ (µg/ml)** |
| --- | --- |
| FST | 77.83 ± 2.12 |
| FHIC | 68.11 ± 1.52 |
| FHIC-PNP | 22.09 ± 1.3 |
